# Accurate optimization technique for phase-gradient metasurfaces used in compact near-field meta-steering systems

**DOI:** 10.1038/s41598-022-08143-x

**Published:** 2022-03-08

**Authors:** Khushboo Singh, Muhammad U. Afzal, Karu P. Esselle

**Affiliations:** University of Technology, School of Electrical and Data Engineering, Sydney, 2007 Australia

**Keywords:** Engineering, Electrical and electronic engineering

## Abstract

Near-Field Meta-Steering (NFMS) is a constantly evolving and progressively emerging novel antenna beam-steering technology that involves an elegant assembly of a base antenna and a pair of Phase-Gradient Metasurfaces (PGMs) placed in the near-field region of the antenna aperture. The upper PGM in an NFMS system receives an oblique incidence from the lower PGM at all times, a fact that is ignored in the traditional design process of upper metasurfaces. This work proposes an accurate optimization method for metasurfaces in NFMS systems to reduce signal leakage by suppressing the grating lobes and side lobes that are innate artifacts of beam-steering. We detail the design and optimization approach for both upper and lower metasurface. Compared to the conventionally optimized compact 2D steering system, the proposed system exhibits higher directivity and lower side-lobe and grating lobe levels within the entire scanning range. The broadside directivity is 1.4 dB higher, and the side-lobe level is 4 dB lower in comparison. The beam-steering patterns for the proposed 2D compact design are experimentally validated, and the measured and predicted results are in excellent concurrence. The versatile compatibility of truncated PGMs with a low gain antenna makes it a compelling technology for wireless backhaul mesh networks and future antenna hardware.

## Introduction

The growing hope and hype around the Internet of Things (IoT) and constantly increasing demand for ubiquitous coverage with ultra-high-speed data over mobile networks has created a new wave of technological revolution in 5G cellular communication. The mobile backhaul technology, primarily used to feed massive data to the end-users, connects the network hub to the base stations wirelessly. It is the most cost-effective and versatile solution to connect 5G base stations to the core network to enable flexible and easy installation of base stations in ad-hoc networks, supporting large crowd gatherings such as concerts and sports events. These backhaul networks depend on the line-of-sight communication between two fixed antenna terminals with point-to-point data transmission^1^. Any error due to antenna misalignment should be avoided to establish a robust link quality in these networks. Thus, a medium/high-gain 2D beam-steering-enabled antenna system with a planar profile is desirable to ease the deployment and cope with the wind-induced movements in the lampposts and other 5G small cell installation sites. These antennas must have the ability to transmit and/or receive in any arbitrary direction within a large conical region.

Conventional mainstream configurations for beam-steering include mechanically moving a fixed beam antenna on a gimbal and electronically steering beams of phased arrays. The mechanical steering solutions are bulky, and a more sophisticated system needs expensive motors to ensure the reliability of vital data links. Active electronically scanned arrays are the most agile front-end systems, but because of the high-cost active Radio-Frequency (RF) components, their use is limited to high-end applications^2,3^. Due to the limitations associated with traditional beam-steering methods, several unconventional technologies have been investigated, including optical beamformers^4^, liquid crystal-based electronic beam-steering^5^, and metasurface antennas and lenses^6–8^. In a basic comparison, the passive beam-steering antennas (without using active RF components) have certain advantages over active beam-steering antennas. The former is relatively cheaper, has better linearity and scalability, low noise, high power handling capability, broader bandwidth, and low DC power consumption, making them suitable for emerging commercial applications.

**Figure 1:**
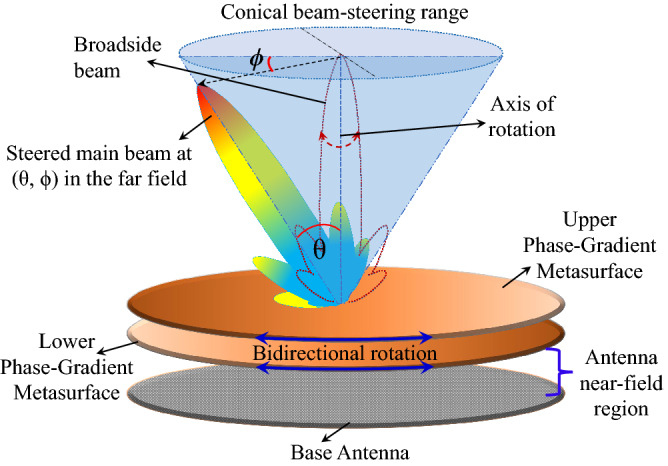
Configuration of classical Near-Field Meta-Steering system.

Near-Field Meta-Steering is a state-of-the-art passive beam-steering antenna technology inspired from the optical-beam scanning principle that is implemented using Risley prisms^9–11^. The concept was first introduced and demonstrated in 2017^12^ and since then has been extended by various research groups^12–16^. The configuration of the classical NFMS system is shown in Fig. 1.

It consists of medium/high-gain base antennas that can create planar phase fronts on the aperture^17^ and a pair of rotating PGMs placed above the base antenna in its near-field region. The first PGM tilts the antenna beam by introducing a phase shift in the antenna electric near field. The beam-tilt angle is controlled through the gradient of phase shift introduced by the PGM. Conventionally, the two PGMs in an NFMS system are identical and introduce the same phase gradient when placed on the antenna with planar phase fronts. In the NFMS systems, depending on its orientation, the second PGM can either increase or decrease the effective phase gradient and hence the antenna beam tilt angle. The base antenna remains fixed, and the two PGMs are co/counter-rotated to steer the antenna beam to any location within a wide 2D conical scanning range. The NFMS antenna technology holds several advantages over conventional steering methodologies. The base antenna being entirely stationary obviates the need for RF rotary joints. Since the entire phase shifting is carried out in the near-field region, the system is extremely low-profile, making it aesthetically pleasing and easier to be mounted on moving platforms. The PGMs can be rotated with a low-power stepper motor, and the total volume of the system is fixed at all times, even when the beam is steered.

The periodic arrangement of physical cells, referred to as supercells, in PGMs generates periodic grating-type lobes. In addition to these grating lobes, the sidelobes appearing within antenna patterns when the beam is steered to larger elevation angles result in signal leakage, which is a concern for most wireless applications. To ensure efficient steering performance, the PGM-pair should be designed strategically. Besides, the sidelobes can be controlled by judiciously tailoring the arrangement of elements in the periodically repeating supercell of the PGMs. The lower metasurface atop the base antenna typically receives a normally incident wave and produces an output beam at an offset angle same as the gradient of the PGM. The design of such PGMs is straightforward and elaborately explained in literature^18–21^. Conventionally, both upper and lower PGMs are designed using the same unit cell-based approach. However, the upper PGM receives an oblique incidence at all times, which calls for a different design and optimization strategy to achieve overall optimal 2D beam-steering performance.

In this work, we propose an innovative and more logical methodology to design and optimize PGMs in an NFMS system by considering the nature of the incident plane wave. The novelty of this work is to design upper PGM with cells having an excellent performance for the obliquely incident electric field. This is the first NFMS design that uses separately characterized cells for the upper and lower PGMs. A Floquet analysis-based Covariance Matrix Adaptation-Evolution Strategy (CMA-ES) optimization similar to the one presented in Ref.^22^ has been used to optimize the lower PGM for normally incident plane waves. The upper PGM is optimized assuming obliquely incident plane wave with an angle of incidence same as the phase-gradient of the first PGM. Two compact beam-steering systems are modeled using a medium/low gain RCA as the base antenna. System-I has a pair of conventionally optimized PGMs (optimized for normal plane wave incidence). System-II has lower PGM optimized for normal incidence, and the upper PGM optimized for oblique incidence. The two systems are then compared based on their steering radiation performance. A prototype of the steering system that has better beam-steering performance is fabricated and measured. The results predicted by simulations in CST MWS are experimentally validated.

## Design methodology

The NFMS system can be operated in three steering modes^12^. One of the modes, where beam is steered in both elevation and azimuth plane is achieved by fixing lower PGM in addition to the base antenna and only rotating the upper PGM. The optimization strategy proposed here, however, can also be implemented without any changes, for other steering modes where both metasurfaces are simultaneously rotated .

### Supercell design approach

**Figure 2:**
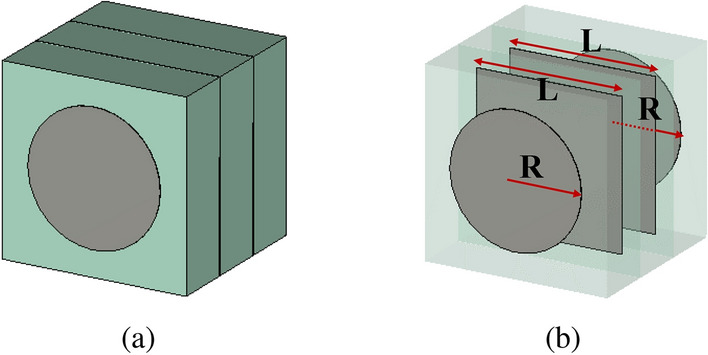
Phase-transforming cell configuration. (**a**) Perspective view. (**b**) Internal configuration of PTC, showing metal patches on four layers.

The PGMs are composed of periodic supercells designed using Phase-Transforming Cells (PTCs) arranged in a pre-defined sequence. The lower PGM is designed to tilt a normally incident plane wave at its input to a $30^\circ$ angle at the output. The upper PGM is designed to transform this obliquely incident plane wave back to the normal at its output. Essentially, the PTCs are the fundamental building block of metasurfaces that control the spatial phase and amplitude variation of electric field passing through them. The configuration of PTCs used in this work is shown in Fig. 2.

Each PTC comprises four metal layers (circular patches on top and bottom layer and square patches in the two middle layers) and three dielectric layers of Taconic TLY-5 ($\epsilon _r = 2.2$, thickness $t=1.5$ mm). The side length of PTC is $d=\lambda _\circ / 3$ ($\lambda _\circ$ is the free space wavelength), which is equal to 5 mm @ 20 GHz.

**Figure 3:**
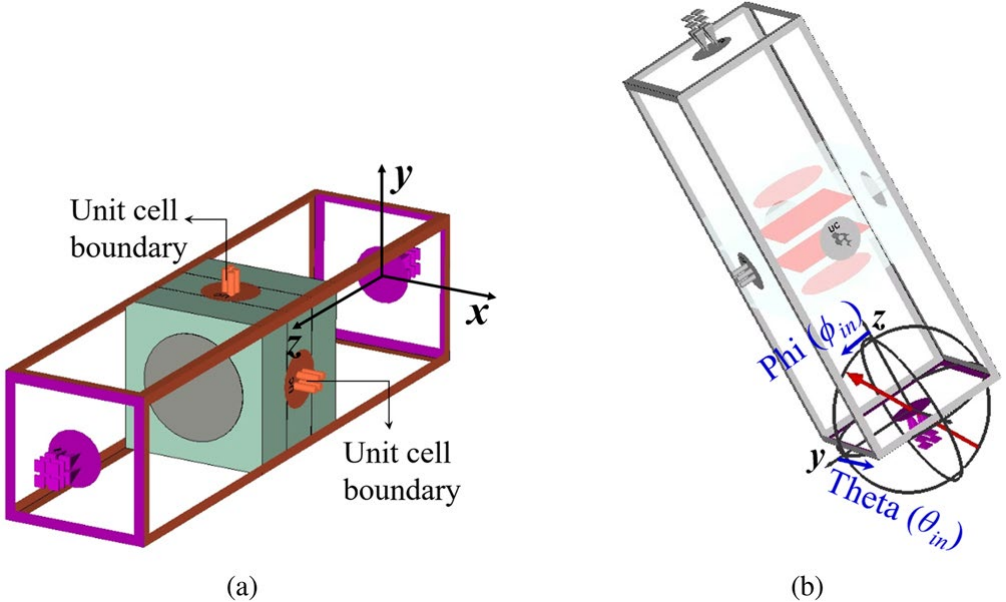
Simulation set-up for a PTC for the upper PGM in a NFMS system. (**a**) Perspective view of a square metal patch PTC with unit cell boundary conditions and (**b**) incidence plane wave direction defined by $\theta _{in}$ and $\phi _{in}$.

For lower PGM, the PTCs are simulated with periodic boundary conditions and normal incidence excitation. For upper PGM, the PTCs are simulated with periodic boundary conditions and oblique incidence excitation, as shown in Fig. 3.

**Figure 4:**
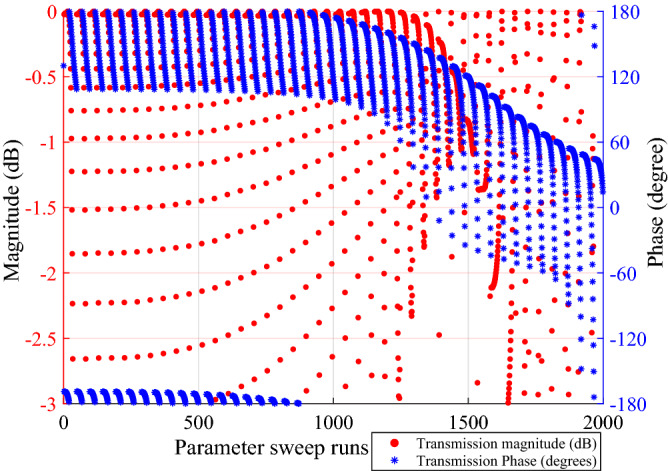
Transmission phases and corresponding transmission magnitudes (greater than − 3 dB) as a function of the parameter sweep on radius and side-length of metallic patches.

The unit cell boundary conditions are applied along *x*- and *y*-directions as shown in Fig. 3a. The incident wave propagation vector shown by the red arrow in Fig. 3b is defined in terms of $\theta _{in}$ and $\phi _{in}$, where $\theta _{in}$ is the oblique elevation angle, and $\phi _{in}$ is the azimuth angle. For lower PGM design, both $\theta _{in}$ and $\phi _{in}$ are zero. For upper PGM design, $\theta _{in}$ is equal to the beam tilt angle of the lower PGM, while $\phi _{in}$ is kept as zero. To accurately choose the constituent elements for the design of PGMs, a parameter sweep is performed on the dimensions of metallic patches in the PTCs for both lower and upper PGMs. The values of transmission magnitudes and corresponding transmission phases are stored in database-I for upper PGM and in database-II for lower PGM. The phase values were normalized in both databases such that a PTC with no patches (i.e., all-dielectric, R = L = 0) produced $0^\circ$ normalized phase shift. Those two sets of data were then used to find the dimensions of the patches that would produce the phase shift required for an actual beam-tilting metasurface at each location. The plot for available transmission phases and corresponding transmission amplitudes (greater than $-3$ dB) as a function of the parameter sweep on radius and side-length of metallic patches in the PTC is provided in Fig. 4. The radius of the circular metal patch is varied from 0.05 mm to 2.4 mm at a step of 0.05 mm and the side-length of the square metal patch is varied from 0.1 mm to 4.8 mm at a step of 0.1 mm. The PTC covers the entire $360^\circ$ transmission phase range with high transmission magnitude.

In a PGM, each periodically repeating supercell cover the phase range from $0^\circ$ to $360^\circ$. For a ${30}^\circ$ beam-tilt, a phase shift $\Delta \phi = 60^\circ$ between adjacent cell in a supercell is calculated from the equation below:^18,23^

1 $$\begin{aligned} \Delta \phi = \frac{2\pi }{\lambda _\circ } d \sin {\theta }, \end{aligned}$$

where *d* is the size of the cell in terms of free-space wavelength. The number of unique PTCs $n = 2\pi /\Delta \phi = 6$. Hence, we select six different unit cells with high transmission and desired transmission phases from database-I to design supercells for upper PGM. The other six desired unit cells were selected from database-II to design supercells for lower PGM. Table 1 shows the unique phase values ($\Phi _i$), here i = 1, 2, $\ldots$, n, ideally required to design the two PGMs for 2D beam steering, along with the corresponding closest phase shifts available in the two databases. The table also provides the corresponding transmission and reflection magnitudes as well as the dimension of metallic patches for each PTC used in the design of upper and lower PGM. The transmission and reflection magnitudes are very critical for near-field phase transformation metasurfaces because they are to be placed in a close proximity to the base antenna. Hence, the generic configuration of the PTCs is of immense importance because it determines the maximum phase range together with high transmission magnitude that can be achieved. The proposed PTC with a combination of circular and square printed metal patches outperformed the PTCs with patches that are all square and those with patches that are all circular, hence are used in this work. The efficiencies of constituent PTCs is also listed in Table 1 for both lower and upper PGMs.

**Table 1:** Transmission and reflection characteristics of the PTCs with respect to patch dimension used in upper and lower PGM design.

PGM	Desired phase shifts	Available phase shifts	Patch dimensions $L_{n}$ (mm), $R_{n}$ (mm)	Transmission magnitude (dB)	Reflection magnitude (dB)	Efficiency $\eta$ of PTCs ($\%$)
Upper PGM	180	180	$L_{1} = 3.5$ , $R_{1} = 2$	− 0.47	− 11.52	94.9
	240	236	$L_{2} = 3.8$ , $R_{2} = 2.15$	− 0.68	− 9.98	90.6
	360	298	$L_{3} = 4.1$ , $R_{3} = 2.25$	− 0.44	− 10.21	92
	0	0	$L_{4} = 0.1$ , $R_{4} = 0.05$	− 0.12	− 15.56	98.4
	60	60	$L_{5} = 2.9$ , $R_{5} = 0.95$	− 0.1	− 16.86	98.5
	120	120	$L_{6} = 3.3$ , $R_{6} = 1.9$	− 1.01	− 8.76	89.29
Lower PGM	180	180	$L_{1} = 3.8$ , $R_{1} = 1.95$	− 0.17	− 14.86	96.9
	240	236	$L_{2} = 3.9$ , $R_{2} = 2.15$	− 0.14	− 14.86	93.8
	360	298	$L_{3} = 4$ , $R_{3} = 2.25$	− 0.008	− 27.27	97.1
	0	0	$L_{4} = 0.1$ , $R_{4} = 0.05$	− 0.02	− 22.83	99.6
	60	60	$L_{5} = 3$ , $R_{5} = 0.95$	− 0.36	− 10.99	95.7
	120	120	$L_{6} = 3.3$ , $R_{6} = 1.9$	− 0.44	− 10.07	− 95.5

Usually, the phase is wrapped back to $0^\circ$ at the edge of the supercell, and this pattern repeats. The EM simulation tool (CST MWS) uses periodic boundary conditions to simulate the periodicity of the supercells. For our analysis we shifted the phase-wrapping point from edges to the middle of the supercell.

**Figure 5:**
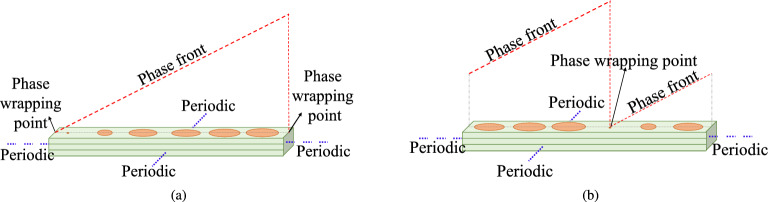
Supercells for $30^\circ$ beam-tilt with (**a**) phase wrapping at the edges, (**b**) with phase wrapping somewhere in the middle.

Figure 5 shows two supercells, one with phase wrapping on the edges and the other with the phase wrapping in the middle. It should be noted that a shift in the absolute phase values does not affect the overall performance of the metasurfaces as long as the relative phase difference between the adjacent cells is kept the same.

### Optimization of PGMs in NFMS system

Floquet based optimization is a physics-oriented approach that acquires its concept from Floquet mode analysis of periodic structures^24,25^. The PGMs are designed by periodically repeating the supercells in a 2D plane. A PGM essentially mimics the behavior of a blazed grating, and the supercells are analogous to one period of that diffraction grating. The number and location of transmitted diffraction modes are obtained from the diffraction grating equation expressed as:

2 $$\begin{aligned} L(\sin \theta _{m} - \sin \theta _{i}) = m\lambda , \end{aligned}$$

where $\theta _{m}$ is the angle of diffraction, $\theta _{i}$ is the angle of incidence, *m* is the order of diffraction, $\lambda$ is the wavelength, and *L* is the length of the supercell (the period of the grating). This concept is extended to optimize a smaller periodic supercell to enhance performance of a large metasurface, thus reducing computational cost and saving time and resources^22^.

We simulate the supercells with periodic boundary conditions and Floquet port excitation in CST MWS. For lower PGM, the supercell was excited with a broadside TE(00) mode propagating along the *z*-axis. For upper PGM, the supercell was excited with an obliquely incident TE(00) mode. The orientation of oblique incidence is defined in terms of elevation angle $\theta _{in}$, which is equal to the beam tilt obtained from the bottom PGM (here $\theta _{in} = 30^\circ$) and azimuth angle $\phi _{in} = 180^\circ$. This simulation setup mimics the orientation of PGM-pair in an NFMS system when $\psi _1 = 0^\circ$, $\psi _2 = 180^\circ$ ($\psi _1 - \psi _2 = 180^\circ$) and the beam is in the broadside direction. Both supercells support ten propagating TE modes, including five transmitting and five reflecting modes. The radiation pattern of finite-aperture metasurfaces made of 8 repetitions along *x-axis* was generated using an array calculator in CST by implementing pattern multiplication to the far-field of a single supercell. An excellent correlation exists between the magnitudes and directions of the Floquet modes predicted by Floquet analysis of supercell and magnitudes and directions of the lobes in the far-field pattern obtained using the supercell simulation together with array calculation. Floquet space analysis of the two supercells reveals that only three out of five propagating modes in the transmission region are significant while the rest are evanescent. The knowledge derived from this analysis presents us with a possibility of controlling the side-lobes and grating lobes by optimizing the coupling of incident energy in the propagating modes^22^.

The dimensions of the circular and square metal patches in both lower and upper supercells are optimized to bring 9 undesired modes (UDMs) below $-35$ dB and simultaneously maintain the desired mode (DM) above $-0.1$ dB using the CMA-ES algorithm in the CST optimizer. The fitness function (FF) in Eq. (3) is defined as a weighted sum of objectives mentioned above to selectively suppress the unwanted grating lobes:

3 $$\begin{aligned} FF = [w_m\{\max (0,(-0.1- DM))\}]^2 + \sum _{i=1}^9[w_i\{\max (0,(UDM -(-35)))\}]^2, \end{aligned}$$

where $w_m$ is the weight associated with the desired mode (*DM*), and $w_i$ are the weights associated with the undesired modes (*UDM*). The value of $w_m$ is fixed to 20, and $w_i$ can vary between 1 to 19. To formulate a single fitness function that ensures high transmission of desired mode while simultaneously suppresses the transmission of spurious grating lobes, the maximum weight is assigned to increase the desired mode above $-0.1$ dB and the undesired modes are assigned weights proportional to their magnitude in the initial design to reduce them below $-35$ dB. The weights assigned to undesired modes must never be greater than that assigned to the desired mode since the primary objective is to have maximum energy directed to the desired mode and only then ensure suppression in undesired modes. The weights are higher for the UDMs with higher magnitude and vice-versa.

Since the optimization variables are continuous, the optimization algorithms inbuilt in CST work reasonably well. The initial dimensions from Table 1 are used as a seed for the algorithm in both upper and lower PGM optimization. The CMA-ES algorithm minimizes the fitness function defined in Eq. (3) in both lower and upper PGM optimization. The algorithm was stopped manually when there was no improvement (reduction) in fitness function for last 50 runs. The simulation time for the supercells is approximately 7 min and 21 s. The upper supercell optimization was stopped after 684 function evaluations. The lower supercell completed 853 function evaluations before it was terminated manually. The dimensions of the initial and optimized supercell for both upper and lower PGMs are provided in Table 2.

**Table 2:** Dimensions of supercells and grating lobe levels before and after optimization (*mm*).

Supercells	Initial dimensions (mm)		Optimized dimensions (mm)		Grating lobe level before optimization (dB)	Grating lobe level after optimization (dB)
Lower PGM	L1 = 3.8 L2 = 3.9 L3 = 4. L4 = 0.1 L5 = 3 L6 = 3.4	R1 = 1.9 R2 = 2.15 R3 = 2.15 R4 = 0.05 R5 = 0.95 R6 = 1.9	L1 = 3.44 L2 = 3.91 L3 = 4.08 L4 = 0.09 L5 = 3.05 L6 = 3.28	R1 = 1.95 R2 = 2.13 R3 = 2.23 R4 = 0.05 R5 = 0.98 R6 = 1.93	$T_0 = -13.5$ dB ($0^\circ$) $T_{-1} = -14.01$ dB ($-30^\circ$) $T_{+1} = -0.59$ dB ($30^\circ$) $T_{-2} = -28.18$ dB ($-60^\circ$) $T_{+2} = -22.36$ dB ($60^\circ$)	$T_0 = -30.57$ dB ($0^\circ$) $T_{+1} = -0.13$ dB ($30^\circ$) $T_{-1} = -30.07$ dB ($-30^\circ$) $T_{+2} = -29.9$ dB ($60^\circ$) $T_{-2} = -31.65$ dB ($-60^\circ$)
Upper PGM	L1 = 3.5 L2 = 3.8 L3 = 4.1 L4 = 0.1 L5 = 2.9 L6 = 3.3	R1 = 2 R2 = 2.15 R3 = 2.25 R4 = 0.05 R5 = 0.95 R6 = 1.9	L1 = 3.27 L2 = 3.93 L3 = 4.11 L4 = 0.1 L5 = 3.07 L6 = 2.93	R1 = 2.12 R2 = 2.1 R3 = 2.18 R4 = 0.04 R5 = 0.87 R6 = 1.93	$T_0 = -0.63$ dB ($0^\circ$) $T_{-1} = -14.16$ dB ($-30^\circ$) $T_{+1} = -12.43$ dB ($30^\circ$) $T_{-2} = -32.49$ dB ($-60^\circ$) $T_{+2} = -28.09$ dB ($60^\circ$)	$T_0 = -0.169$ dB ($\theta =0^\circ$) $T_{-1} = -36.74$ dB ($-30^\circ$) $T_{+1} = -30.11$ dB ($30^\circ$) $T_{+2} = -32.15$ dB ($60^\circ$) $T_{-2} = -30.35$ dB ($-60^\circ$)

**Figure 6:**
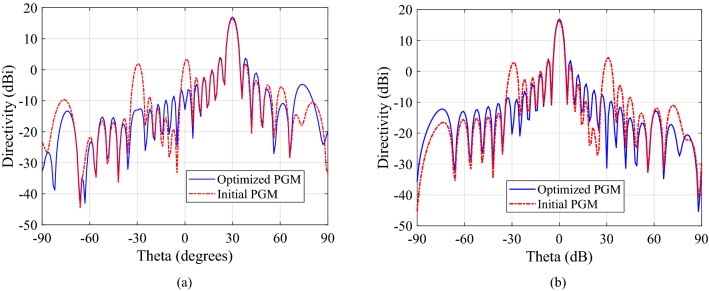
(**a**) Comparison between the steered radiation pattern predicted using CST array calculator for $12\lambda _{0}\times 12\lambda _{0}$ aperture of optimized lower PGM and initial lower PGM when excited with a normally incident plane wave and (**b**) optimized upper PGM and initial upper PGM when excited with an oblique incidence ($\theta _{in} = 30^\circ$ and $\phi _{in} = 180^\circ$).

**Figure 7:**
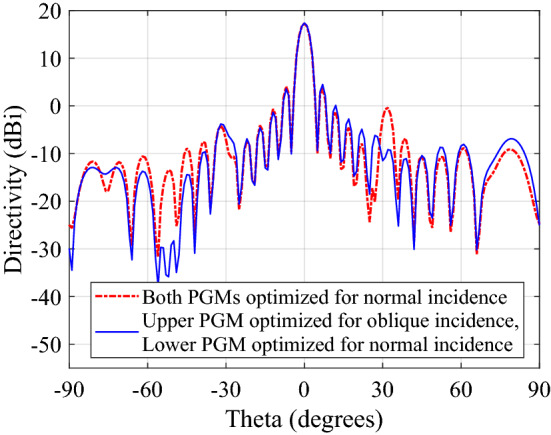
Comparison between the broadside radiation pattern predicted using CST array calculator for a pair of conventionally optimized PGMs for normal incidence and a pair of PGMs where the upper PGM is optimized for oblique incidence while lower PGM is optimized for normal incidence. The PGMs pairs are aligned such that $\psi _1 = 0^\circ$ and $\psi _1 = 180^\circ$.

The magnitude of grating lobes before and after optimization are also provided in Table 2, for both upper and lower PGMs. CST array calculator is used to generate the far-field pattern for a metasurface aperture of ($12\lambda _{0}\times 12\lambda _{0}$) designed by repeating the two supercells six times along *x*-direction and 36 times along *y*-direction. The predicted radiation pattern for initial and optimized lower PGM is compared in Fig. 6a. Figure 6b compares the predicted broadside radiation patterns of initial and optimized upper PGMs. We observe that the grating lobes in the predicted steered radiation pattern of lower PGM and the broadside radiation pattern of upper PGM are higher for the initial unoptimized supercell than the optimized supercell. However, the above simulation setups assumes an ideal scenario when the incident plane wave is entirely uniform. In practice, the PGMs exists in pair, placed above (and close to) the base antenna, in a NFMS system. To study the actual performance, the supercell of upper PGM optimized for oblique incidence was rotated $180^\circ$ and placed above the supercell of lower PGM optimized conventionally (assuming normal incidence) at a distance of $\frac{\lambda _0}{/}{4}$ such that it represents the PGM-pair orientation when $\psi _1 = 0$ and $\psi _2 = 180^\circ$. The setup was excited with a normal plane wave incidence. The broadside radiation pattern predicted using the CST array calculator is compared with the pattern of a similarly arranged pair of conventionally optimized supercells (assuming normal incidence) in Fig. 7. In this way, we get a more accurate prediction of the radiation pattern for the full PGM-pair when placed in the near field of a high gain antenna with uniform plane wave output. It is observed that the radiation pattern for a PGM-pair has lower grating lobes when upper PGM is optimized for oblique incidence and lower PGM is optimized for normal incidence. It is imperative to optimize the PGMs in an NFMS system strategically to enhance overall system performance.

Optimizing electrically large PGMs involves highly non-linear objective functions that exhibit an epistatic behavior due to strong mutual coupling and other propagation effects. Such design problems require a full-wave simulation-based derivative-free optimization approach. Evolutionary Algorithms (EAs) are a class of derivative-free optimization with superior exploration skills to search intractably large spaces. They can successfully achieve an optimal solution with a high probability for complex, multi-dimensional problems, operate with continuous, discrete as well as mixed parameters, are conducive to parallel computation, which significantly reduces the optimization run time by distributing the task among multiple computers. Their outcome is independent of the initial guess (provided the algorithm can explore and exploit the entire search space). Some of the widely used optimization methods in the field of Antenna and Propagation include Genetic Algorithm (GA), Particle Swarm Optimization (PSO), CMA-ES. GA and PSO optimization methods are susceptible to hyperparameters of the algorithms and require tedious parameter tuning, which is a challenging task. A better choice of the algorithm’s hyperparameters adapted to each function and dimension can seriously influence the final result. Since the CMA-ES method does not require tedious parameter tuning, the choice of the strategy to be adopted while setting the internal parameters is entirely automated. Therefore, it is more convenient than algorithms such as PSO and GA and hence is chosen in this work.

## Design example

We demonstrated the complete system by developing a compact NFMS system. The base antenna is a resonant cavity antennas (RCAs), which is also referred to as leaky-wave antenna, Fabry–Perot antenna and electromagnetic bandgap (EBG) antenna in the literature^17,26^. RCAs are simple, efficient, and planar antennas with high directivity. They can be designed for both liner and circular polarizations. Low-profile medium-to-high gain RCAs find several applications in high-speed local wide-area networks and front-end antennas for point-to-point communication systems, such as commercial access and backhaul networks^1,17^. We uses a compact, strongly truncated RCA as a source of EM radiation that is known for its planar phase fronts since they have reasonably uniform phase in their aperture electric field. The base antenna is linearly polarized with the electric field parallel to the y-axis.

### Classical uniform-superstrate compact RCA design

**Figure 8:**
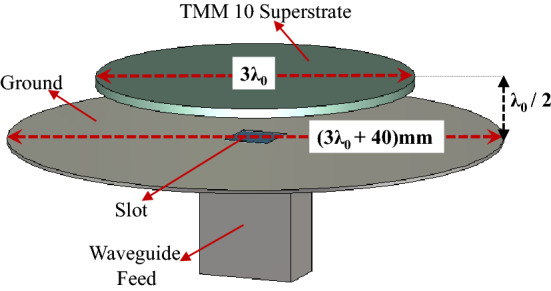
Configuration of a single layer uniform substrate RCA.

**Figure 9:**
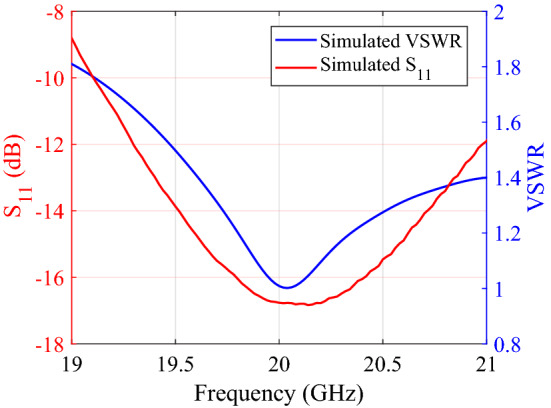
Predicted $S_{11}$ and VSWR for the truncated RCA.

A classical single layer RCA with a uniform dielectric superstrate composed of a circular disc of Rogers TMM 10 placed a half-wavelength ($\lambda _0/2$) above a circular fully-reflective ground plane as shown in Fig. 8 is considered. The cavity between ground and superstrate is excited with a slot (of size 6 mm $\times$ 6 mm) in a rectangular waveguide at 20 GHz. The diameter of the ground plane is deliberately kept larger to ensure that the antenna can be securely mounted on the AUT stage in the anechoic chamber. The superstrate’s diameter is kept as $3\lambda _0$, where $\lambda _0$ is the free space wavelength at 20 GHz. The relative permittivity of Rogers TMM 10 dielectric superstrate is $\varepsilon _{r} = 9.8$. The thickness of the superstrate is $t = 0.25\lambda _{g} = 0.25\times \frac{\lambda _{0}}{\sqrt{\varepsilon _{r}}}$ at 20 GHz operating frequency. The directivity of the RCA increases with the increase in the permittivity of the superstrate due to an increase in the reflectivity. The boresight directivity of this classical single layer type RCA is approximately 16.8 dBi. The realized gain is 16.2 dB. Hence, the predicted radiation efficiency of the base antenna is $96.42\%$. The predicted $S_{11}$ and VSWR for the truncated RCA is also plotted in Fig. 9.

### Compact beam-steering RCA system design

**Figure 10:**
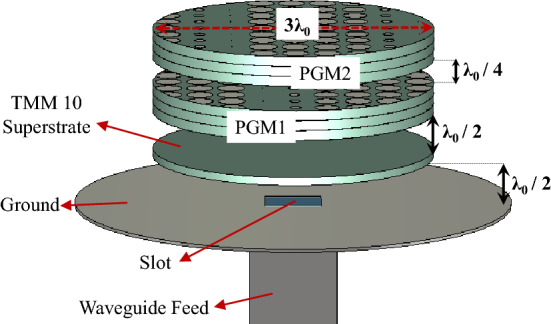
Compact NFMS system design.

The configuration of the compact NFMS system with three-dimensional model is shown in Fig. 10. It has a strongly truncated classical RCA having circular disc of uniform dielectric superstrate made of Rogers TMM 10. A pair of truncated PGMs with an aperture of diameter $3\lambda _{0}$ (same as the RCA superstrate) is placed half wavelength above the RCA aperture. The RCA produces an electric field with a fairly uniform phase in the near-field, which creates a broadside beam in the far-field. The relative rotation between the PGM-pair causes the beam to steer in the 3D conical volume/space, and the complete steering process is explained in brief in Ref.^22^ and in detail in Ref.^12^. When the two identical PGMs are aligned such that $\psi _1 = 0^\circ$ and $\psi _2 = 180^\circ$, where $\psi _1$ and $\psi _2$ are the orientation angles of the two PGMs, the beam is in the broadside direction. If the lower metasurface is fixed at $\psi _1 = 0$ and $\psi _2$ is varied from $180^\circ$ to $0^\circ$, the azimuth angle decreases according to $\phi = \psi /2$, while the elevation angle gradually increases to $\theta _{max}$ for a certain value of $\psi _2$ (can be calculated using of phase method in Ref.^27^), beyond which the beam goes in the lower invisible hemisphere until $\psi _2 = 0^\circ$. It is pertinent to mention here that the base antenna with a single PGM can only steer the beam in the azimuth plane. However, a pair PGMs increase the degree of freedom and provide more control over the beam location. A complete 3D scanning can be achieved by co-/counter-rotation of the PGM pairs, and the beam can be steered in both azimuth and elevation planes.

## Results

We designed two compact beam-steering systems of same dimensions as specified in Fig. 10, namely, System-I and System-II. System-I is composed of compact RCA and a pair of PGMs optimized conventionally (considering normal incidence). System-II comprises of compact RCA and a pair of optimized PGMs where upper PGM is optimized for oblique incidence and lower PGM is optimized for normal incidence.

### Comparing two compact beam-steering systems

**Figure 11:**
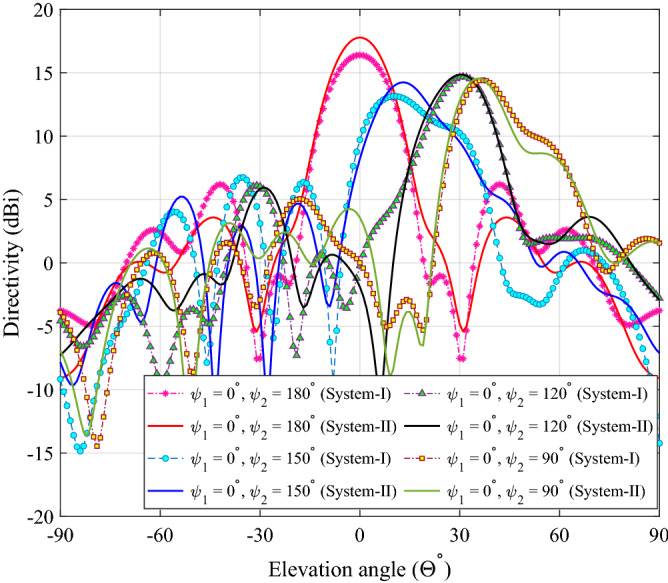
Elevation plane radiation pattern cuts for each rotation of upper PGM ($\psi _1 = 0$ and $\psi _2$ is varied) in the compact NFMS system.

**Figure 12:**
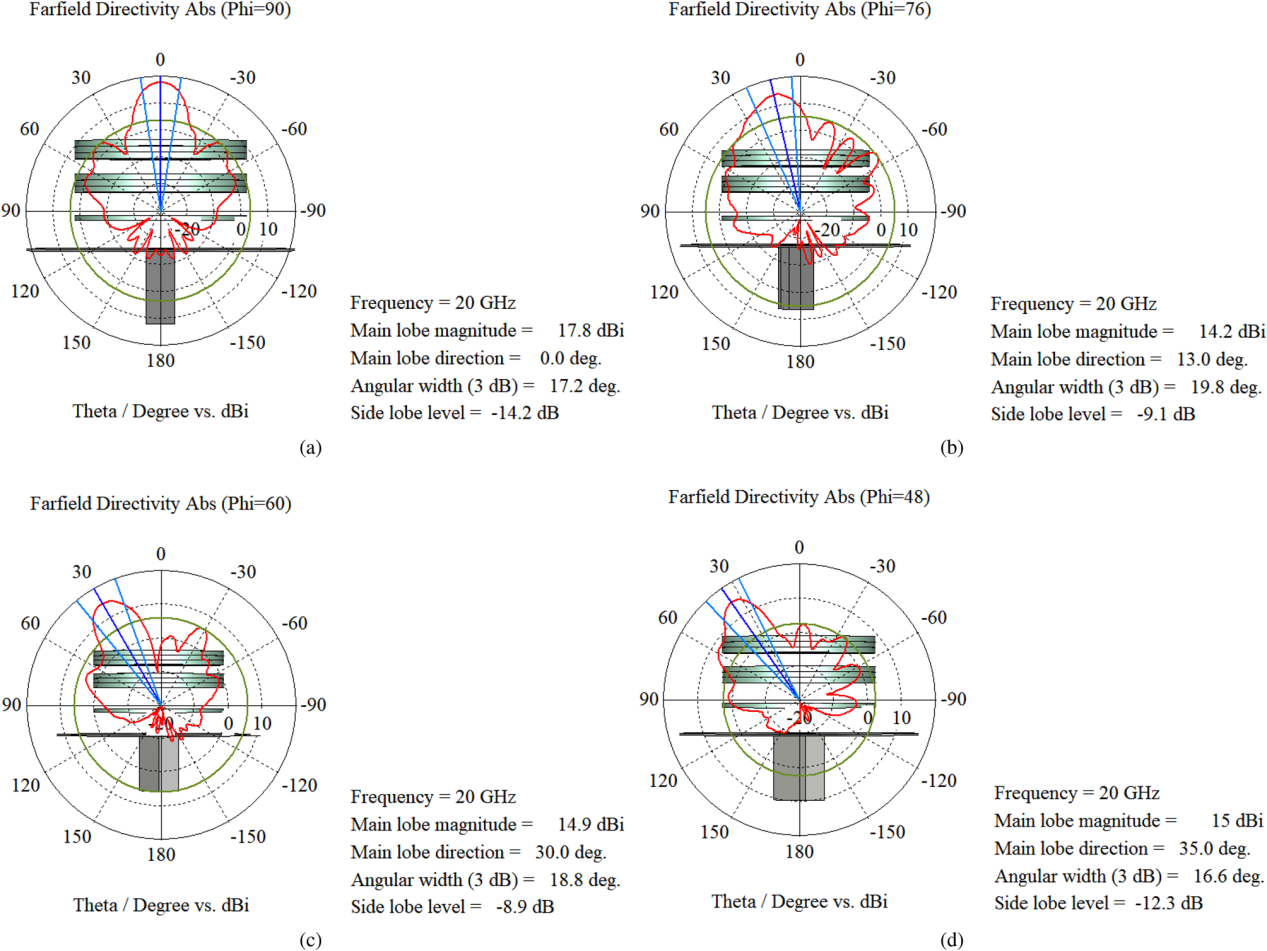
1D polar plots for three different orientations of upper PGM when lower PGM is fixed ($\psi _1 = 0$). (**a**) $\psi _2 = 180^\circ$ (**b**) $\psi _2 = 150^\circ$ (**c**) $\psi _2 = 120^\circ$ and (**d**) $\psi _2 = 90^\circ$.

**Figure 13:**
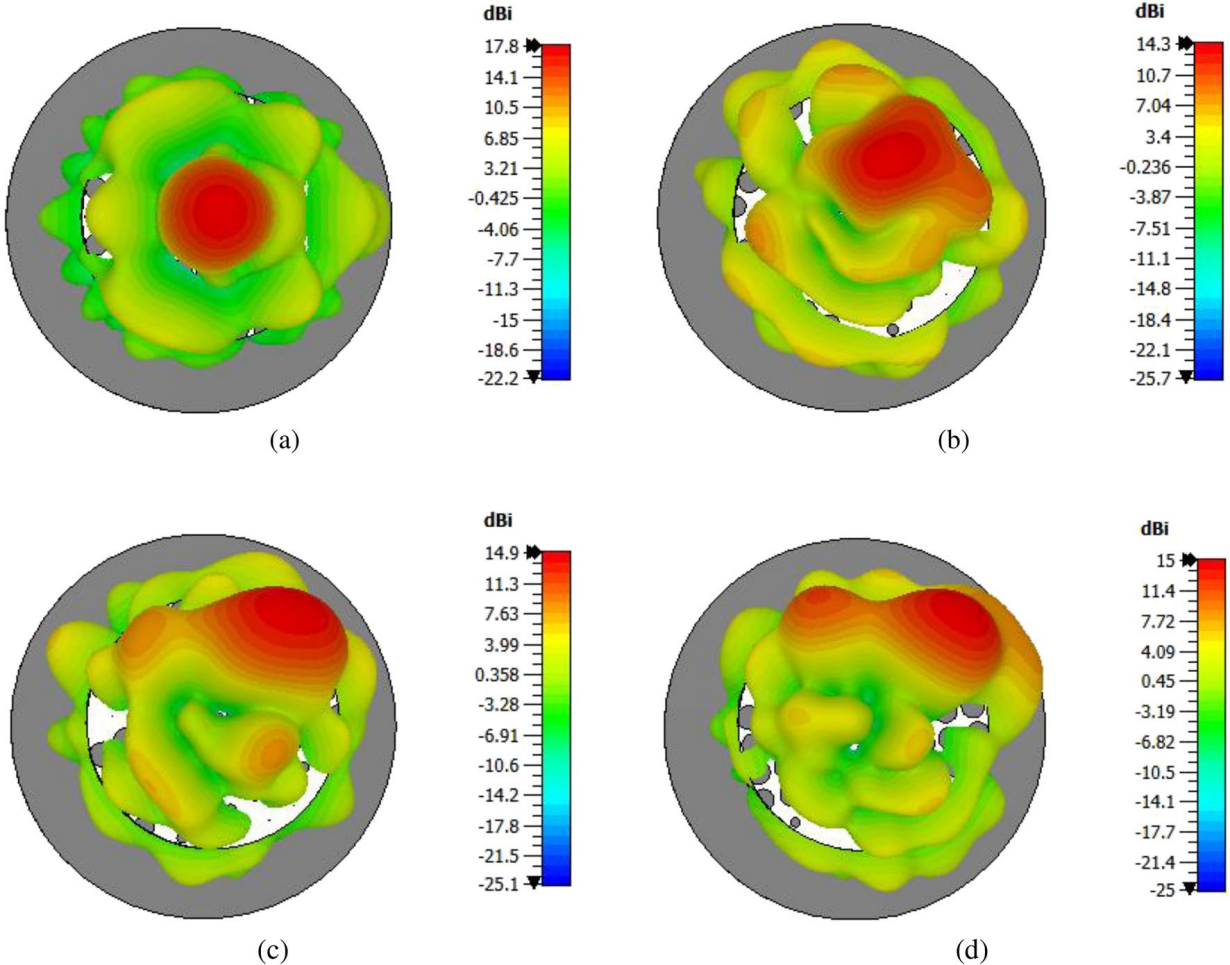
3D radiation pattern for compact NFMS System (front view) when $\psi _1 = 0^\circ$ at all times and $\psi _2$ is varied: (**a**) $\psi _2 = 180^\circ$, (**b**) $\psi _2 = 135^\circ$. 3D radiation pattern for compact NFMS System (front view) when $\psi _1 = 0^\circ$ at all times and $\psi _2$ is varied: (**c**) $\psi _2 = 120^\circ$ and (**d**) $\psi _2 = 90^\circ$.

The elevation pattern cuts for System-I and System-II are compared in Fig. 11 for several orientations of upper PGM when lower PGM is fixed at $\psi _1 = 0^\circ$. For System-I, when the beam is steered away from broadside ($\Theta = 0^\circ$) to elevation angles $13^\circ$, $30^\circ$ and $35^\circ$, the directivity changes from 16.4 dBi, to 13.2 dBi, 14.7 dBi and 14.4 dBi and the side-lobe level changes from $-10.2$ dB to $-6.4$ dB, $-8.6$dB and $-9.1$ dB, respectively. For System-II, the directivity changes from 17.8 dBi, to 14.3 dBi, 14.9 dBi, and 15 dBi, and the side-lobe level changes from $-14.2$ dB to $-9.1$ dB, $-8.9$ dB, and $-12.3$ dB, when the beam is steered to elevation angles $13^\circ$, $30^\circ$ and $35^\circ$, respectively. We observe that System-II has higher directivity and lower SLL for all beam locations, hence we select System-II for further study and final prototype. We then characterize System-II based on steering angle, sidelobes, and directivity. Fig. 12 shows 1D polar plots of radiation patterns for four different orientations (corresponding to four different beam locations) for System-II. Figure 13 shows the 3D patterns for the same four orientations.

### Fabricated prototype

**Figure 14:**
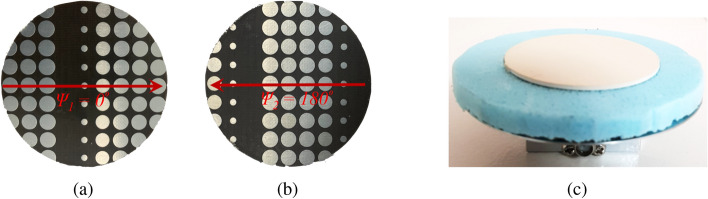
Fabricated prototype of (**a**) Lower phase-gradient metasurface, (**b**) Upper phase-gradient metasurface, (**c**) Single-layer uniform-superstrate resonant cavity antenna. The diameter of both metasurfaces and RCA superstrate is $3\lambda _0$.

The prototypes for the PGM pair of System-II along with the single-layer uniform-superstrate RCA were fabricated as shown in Fig. 14.

The fabricated components are assembled into compact near-field meta-steering system whose steering performance is measured for several configurations to validate numerically predicted results. The primary objective of the proposed design methodology is to demonstrate the steering of antenna beam in both azimuth and elevation planes, with reduced side-lobes and grating lobe levels and simultaneously maintain a fairly stable directivity.

### Experimental results for RCA

**Figure 15:**
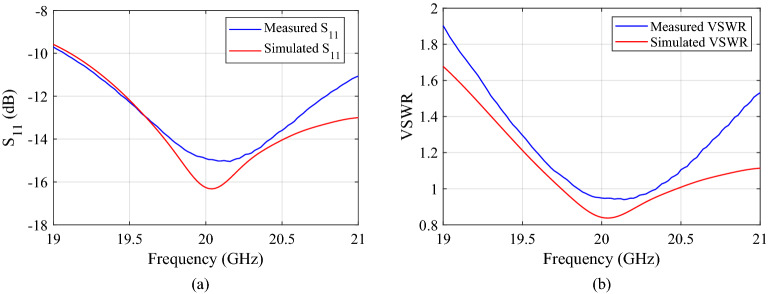
Comparing measured and predicted $S_{11}$ and VSWR of the base antenna (truncated single layer RCA).

We first measure the performance of classical RCA prototype and compare with the simulated results as shown in Fig. 15.

The measured and simulated results are in good agreement. The antenna exhibits a reflection less than $-10$ dB from 19 GHz to 21 GHz and has good matching in the operating band. The VSWR is below 1.9 dB from 19 to 21 GHz.

### Experimental results with one PGM

**Figure 16:**
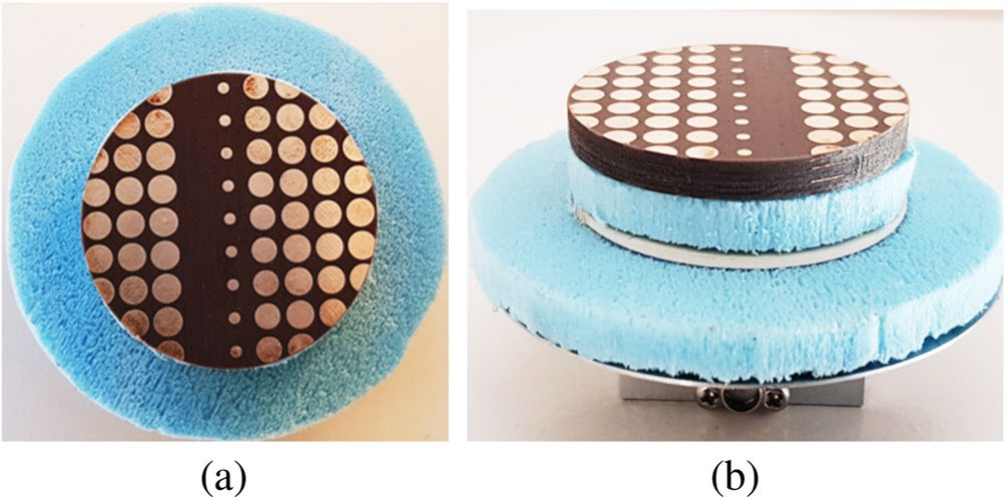
First antenna measurement assembly of RCA and lower PGM (**a**) Top view and (**b**) side view.

**Figure 17:**
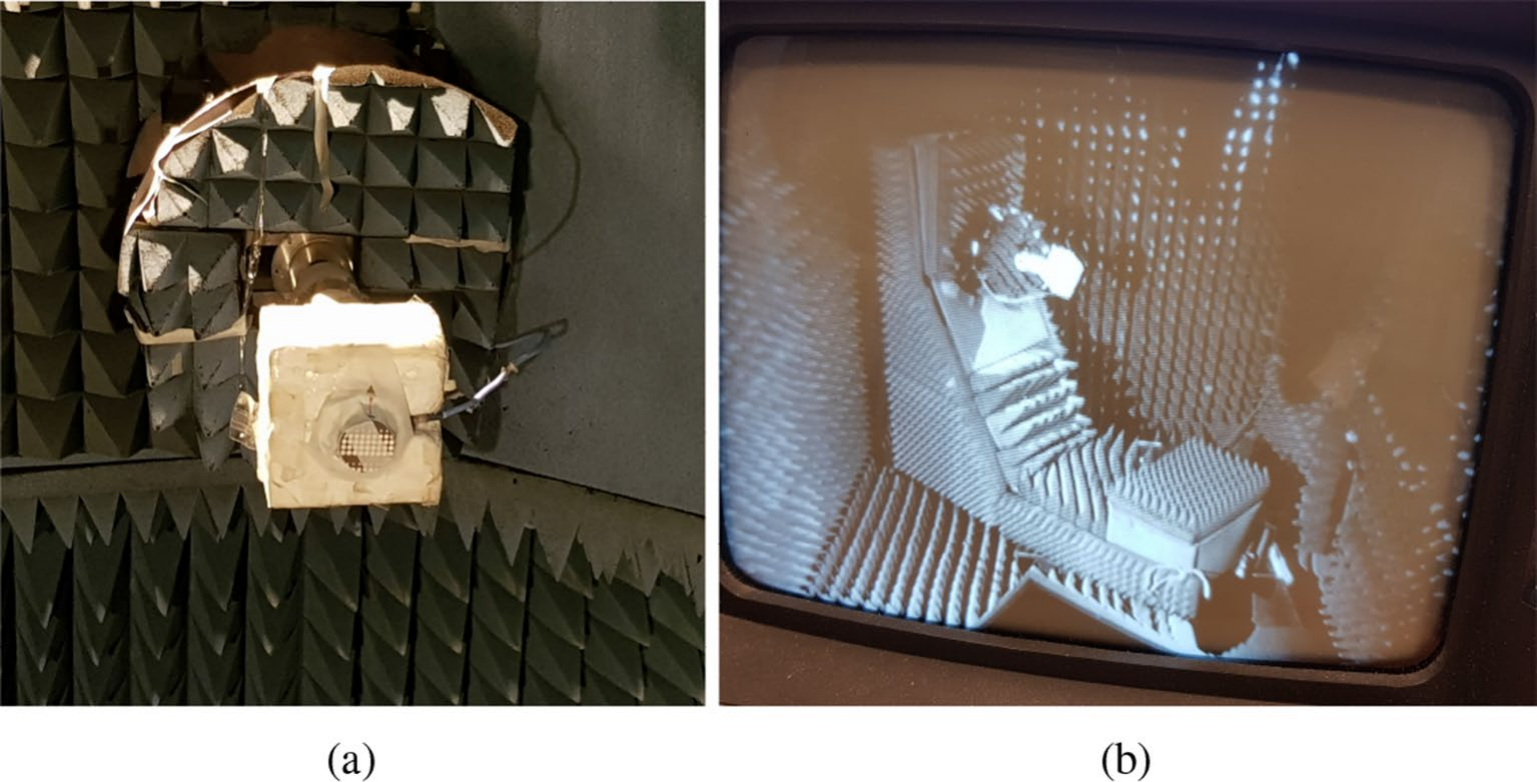
Photos taken inside the anechoic chamber at Australian Antenna Measurement Facility (**a**) Device under test setup (**b**) Monitoring the progress of measurement on a computer screen.

**Figure 18:**
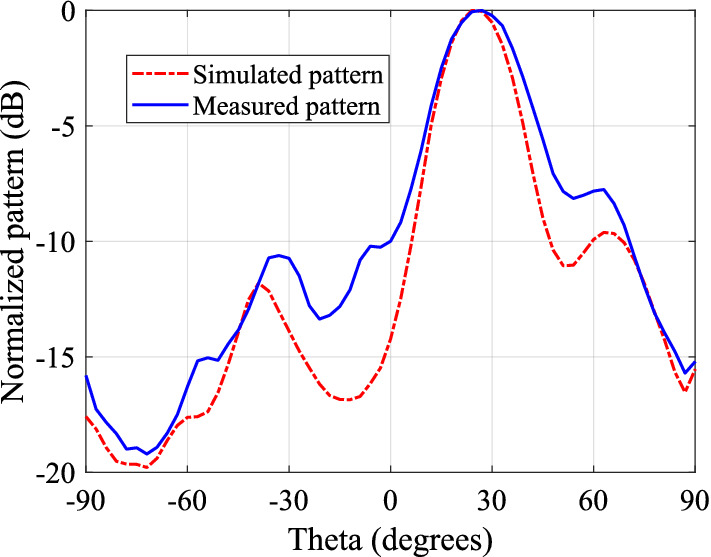
Measured and predicted elevation plane pattern cuts for an assembly of RCA and one PGM.

The first antenna assembly comprises of lower PGM and the RCA as shown in Fig. 16. The PGM is placed half a wavelength (7.5 mm @ 20 GHz) above the RCA that acts as an electromagnetic illuminator with a nearly uniform phase distribution in the aperture. Far-field radiation pattern was measured in an NSI spherical near-field range at Australian Antenna Measurement Facility. The antenna measurement setup in the anechoic chamber is shown in Fig. 17. The normalized measured radiation pattern of RCA with lower PGM oriented at $\psi _1 = 0^\circ$, is compared with the normalized simulated pattern in Fig. 18. The predicted beam-tilt achieved with an assembly of RCA and lower PGM is in close agreement the measured results. The maximum predicted directivity is 14 dBi.

### Experimental results with PGM-pair

**Figure 19:**
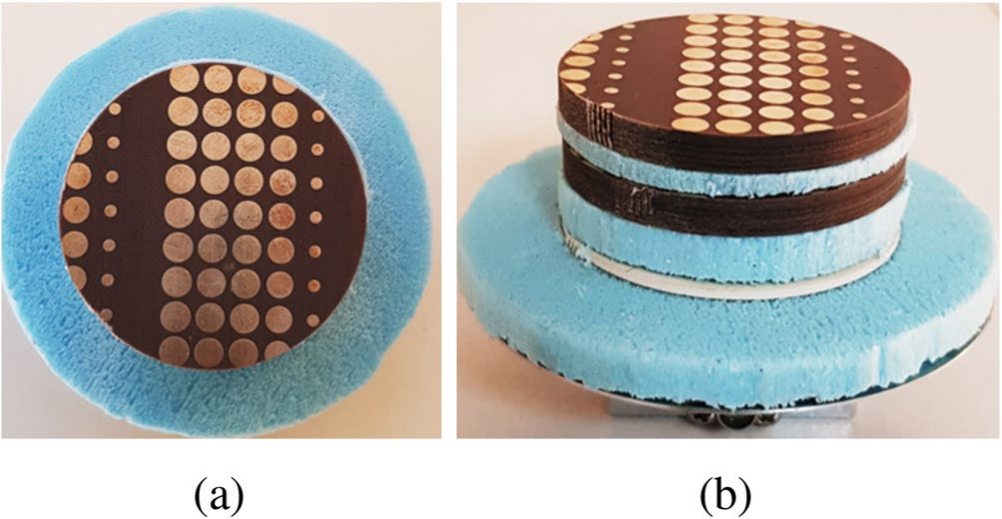
Second antenna measurement assembly of RCA and lower PGM (**a**) Top view and (**b**) side view.

**Figure 20:**
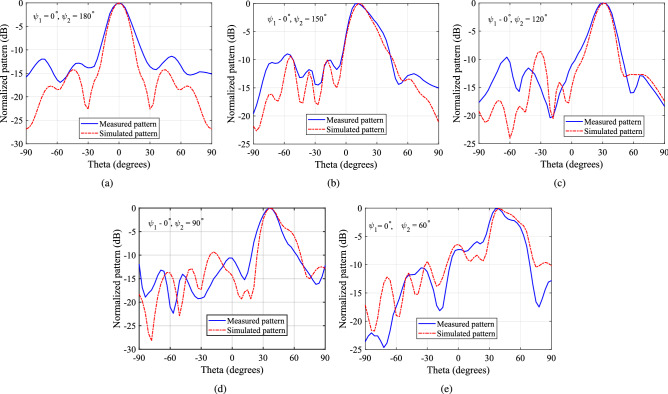
Simulated and measured elevation plane pattern cuts for the case when lower PGM is fixed ($\psi _1 = 0^\circ$) and upper PGM is rotated ($\psi _2$ is varied) from $180^\circ$ to $60^\circ$ at a step of $30^\circ$: (**a**) $\psi _2 = 180^\circ$, (**b**) $\psi _2 = 150^\circ$, (**c**) $\psi _2 = 120^\circ$ (**d**) $\psi _2 = 90^\circ$ and (**e**) $\psi _2 = 60^\circ$. Each cut is taken at the elevation plane in which the pattern peaks.

A photograph of the complete system is shown in Fig. 19. The gap between the two PGMs is quarter wavelength (3.75 mm @ 20 GHz). Spacing in all measurement setups were ensured using Styrofoam layer, which were measured manually and were accurate to about 0.2 mm. The lower PGM was aligned to the x-axis ($\psi _1 = 0^\circ$), while the upper PGM was physically rotated at steps of $-30^\circ$, starting from $180^\circ$ to $60^\circ$ i.e., $180^\circ$, $150^\circ$, $120^\circ$, $90^\circ$ and $60^\circ$. Far-field patterns were measured at each step. Elevation plane pattern cuts measured at elevation planes containing the beam peak are compared with the simulated elevation pattern cuts in Fig. 20. There is a reasonable agreement between the predicted and measured radiation pattern results. The slight discrepancy can be attributed to the human error involved during the measurement in the chamber.

## Discussion

The beam-steering antennas envisage a critical role in mobile backhaul technology. The electronically scanned antenna systems predominantly rely on active components, are complex and expensive. The NFMS antenna systems are an attractive alternative to traditional lossy beam-steering arrays with nearly equivalent performance with passive components. The absence of active elements such as phase shifters and amplifiers unlocks the potential for use in power-limited mobile environments. NFMS systems made of a base antenna and pair of PGMs use an approach to design both PGMs using cells that have excellent performance for normally incident field, which is not valid for the upper PGM. A Floquet analysis based CMA-ES optimization is implemented to first optimize the lower PGM for normal plane wave incidence and then to optimize upper PGM for oblique plane wave incidence to ensure minimal scattering in unwanted direction because of the coupling between different cells in the PGMs. A compact NFMS designed following the approach indicate ability to steer medium-gain beam to the maximum elevation angle of $\pm 35^\circ$ for a 3 dB reduction in peak directivity. Optimizing the performance of PGMs is a crucial step towards their practical implementation in real-time antenna and RF systems. The proposed optimization approach can also be applied to optimize electrically large PGMs in high-gain NFMS systems. Conventional design and optimization approaches rely on extensive parameter searches and do not account for the near-field interactions that strongly influence the overall system performance. The proposed optimization strategy accounts for the mutual coupling between the neighboring metallic patches. Evolutionary algorithms can elegantly handle complex multi-dimensional problems. Hence, PTCs with complex geometries and a large number of design variables can also be optimized using the proposed methodology in conjunction with CMA-ES algorithm.

## Conclusion

A low-cost compact beam-steering system is developed using a pair of truncated PGMs and a medium gain RCA. PGM-pair is optimized using Floquet based analysis and PSO algorithm. Analysis proved that an NFMS system has better steering performance when upper PGM is optimized for oblique incidence, and lower PGM is optimized for normal incidence compared to when both upper and lower PGMs are optimized for normal incidence. The planar profile of the designed steering system facilitates encapsulation into a cylindrical cavity. The metasurfaces are compact and lightweight and can be easily rotated with a pair of low-power stepper motors. Maximum directivity of the antenna system is 17.8 dBi, and it can be used to scan any elevation angle up to $35^\circ$ for a 3 dB reduction in peak directivity for all azimuth angles. Beyond $35^\circ$ elevation angle, the peak directivity decreases considerably. However, if a directivity as low as 10 dBi is accepted, then the system can be used to scan up to $60^\circ$ elevation angle for all azimuth angles. The predicted radiation efficiency for the proposed NFMS system is $92.5\%$, with the peak directivity of 17.8 dBi and a realized gain of 16.45 dB at 20 GHz. When used as front-end antennas, these steering systems can be controlled remotely to tune the direction in case of any misalignment due to unforeseen circumstances. Since the base antenna is tilt-free and only the upper PGM is rotated, this system does not require any rotary joints.
